# A New Report of Combined Central and Peripheral Demyelination: A Case Report

**DOI:** 10.3389/fneur.2021.730129

**Published:** 2021-11-19

**Authors:** Foziah Alshamrani, Rawan Alyami, Ibrahim Alghanimi, Reef Alajaji, Modhi Alkhaldi, Abdulla Alamri

**Affiliations:** ^1^Department of Neurology, King Fahad University Hospital, Imam Abdulrahman Bin Faisal University, Dammam, Saudi Arabia; ^2^College of Medicine, Imam Abdulrahman Bin Faisal University, Dammam, Saudi Arabia; ^3^Department of Radiology, King Fahad University Hospital, Imam Abdulrahman Bin Faisal University, Dammam, Saudi Arabia

**Keywords:** radiologically isolated syndrome, multiple sclerosis, miller-fisher syndrome, combined central and peripheral demyelinating disease, MRI, GQ1b

## Abstract

Combined central and peripheral demyelination (CCPD) is not encountered frequently in the clinical practice, and it requires a high level of suspicion for diagnosis. We describe a case of a young man who was diagnosed with radiologically isolated syndrome (RIS) after presenting initially with symptoms suggestive of central nervous system (CNS) insult in the form of double vision, slurred speech, left-sided numbness, and unsteadiness. However, on the next day of admission, his neurological examination was remarkable for ataxia, areflexia, and ophthalmoplegia, the typical triad of Miller Fisher syndrome (MFS). After confirming both diagnoses, the final diagnosis of CCPD was made. The challenges one may face to diagnose and treat CCPD urge sharing of similar cases to open the door for further extensive and thorough investigations and to encourage further studies and analysis of available data to come up with consolidated management guidelines for rare disorders.

## Introduction

Demyelinating disorders are usually divided into the central nervous system (CNS) and peripheral nervous system (PNS) demyelination. The presence of both—CNS and PNS-demyelinating disease—in a patient is relatively rare (1). In this case report, our patient was diagnosed with Miller Fisher syndrome (MFS), which is a syndrome characterized by ataxia, ophthalmoplegia, and areflexia (2). Moreover, the patient was diagnosed with radiologically isolated syndrome (RIS) based on the MRI findings that are highly suggestive of multiple sclerosis (MS). MS is a chronic inflammatory demyelinating disease of the CNS characterized by multiple lesions disseminated in time and space (3). Our case is notable for the presence of characteristic MS-demyelinating lesions in a patient newly diagnosed with MFS.

## Case Presentation

A 31-year-old left-handed Saudi man, a heavy smoker, who does not consume substance or alcohol, and has no remarkable medical or family history, was admitted with double vision, slurred speech, left-sided numbness, unsteadiness, and constipation. He had no fever on admission, was fully conscious, and had no symptoms of meningeal irritation, history of contact with sick individuals or antecedent vaccination or infection. Before his presentation, the patient had a 2-week history of left upper limb paroxysmal numbness lasting for only a few seconds and was labeled as a possible case of MS at an outside medical facility after brain and spinal magnetic resonance imaging (MRI). There was a history of unintentional 20-kg weight loss in the past 6 months; however, there were no other constitutional symptoms.

Initial examination findings and vital signs were within normal limits, and gastrointestinal, respiratory, and cardiovascular examinations were normal. On neurological examination, the patient was conscious, alert, and oriented. Visual acuity was 20/16 bilaterally, pupillary reflex was 7 mm and non-reactive bilaterally, and bilateral internuclear ophthalmoplegia with left-sided non-fatigable ptosis were observed. The patient had normal tone and muscle bulk. Power was 5 of 5 in all limbs, and all tendon reflexes were +1, with a normal plantar reflex. There was a decrease in vibration sensation up to the head of the fibula on the left side. Dysmetria was noted bilaterally on the finger-to-nose and heel-to-shin tests and was more profound on the left side. Truncal ataxia was noted, and the patient could only walk with assistance and was unable to perform tandem gait.

Biochemical, hematological, liver, and renal functions, virological (HIV, hepatitis B, hepatitis C, herpes simplex Types 1 and 2, rubella, COVID-19), brucellosis, and toxoplasmosis test results were all negative or within normal ranges. Toxicological screening results were negative. Cerebrospinal fluid (CSF) examination showed the following: white blood cells, 2 cu mm; red blood cells, 70%; albumin, 20 mg/dl; protein, 33 mg/dl, glucose, 83 mg/dl; and serum glucose was 128 mg/dl. Oligoclonal bands (OCB) were detected in the CSF and absent in the serum. CSF culture and encephalitis or meningitis panel were negative. Immunological studies were performed; antinuclear antibody, antiphospholipid IgM and IgG, anti-dsDNA, anticardiolipin IgM and IgG, anti-Sjogren antibody SSA and SSB, and extractable nuclear antigen were all negative.

**Figure 1:**
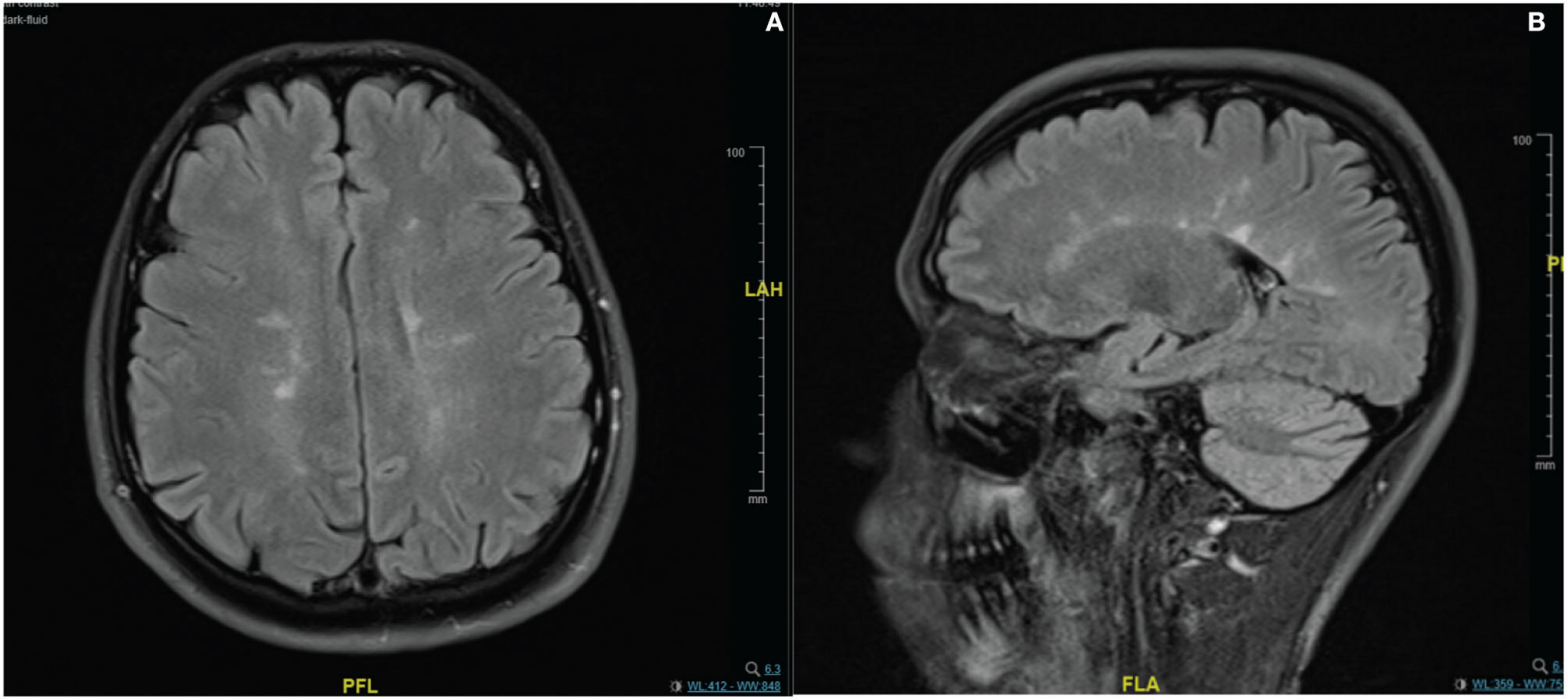
Contrast enhanced MRI brain imaging on the day of admission. Fluid attenuated inversion recovery (FLAIR) sequence **(A,B)**. Showing multiple abnormal high signal intensities involving cortical, juxtacortical, subcortical, and periventricular regions. Some lesions were perpendicularly oriented to the corpus callosum representing Dawson's fingers.

**Figure 2:**
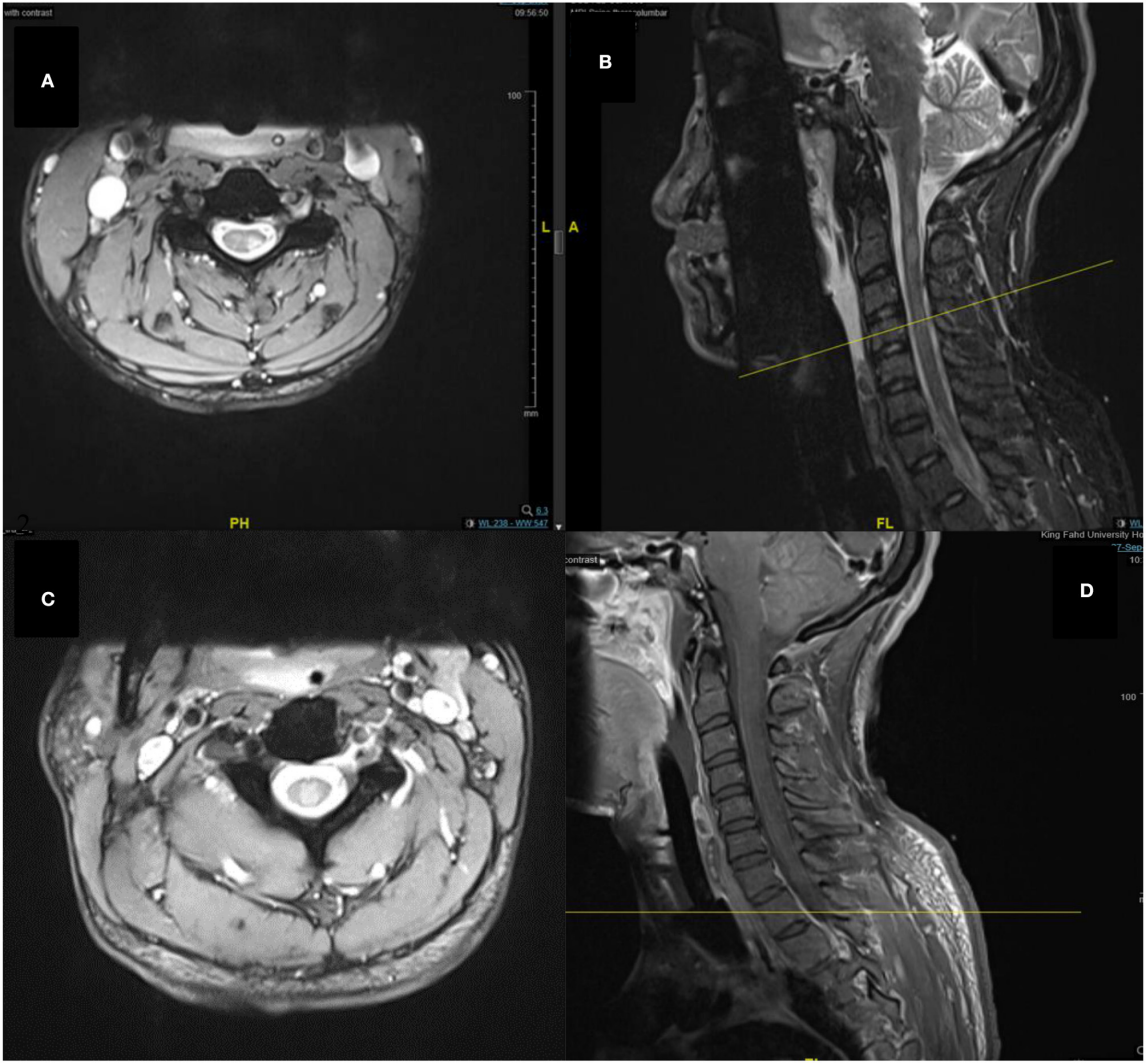
Spine MRI images of T2-weighted **(A)**, sagittal short tau inversion recovery (STIR) **(B)**, T2-weighted **(C)**, and sagittal T1-weighted with contrast **(D)** sequences. Showing multiple abnormal high signal intramedullary lesions at the central, posterior, and lateral aspects of the cervical and thoracic spinal cord.

In the imaging, brain MRI with contrast was performed on the day of admission and showed multiple abnormal high signal intensities involving cortical, juxtacortical, subcortical, and periventricular with one lesion at the left anterior aspect of the pons. Additionally, some lesions were perpendicularly oriented to the corpus callosum representing Dawson's fingers (Figure 1). Spinal cord MRI showed multiple abnormal high signal intramedullary lesions at the central, posterior, and lateral aspects of the cervical and thoracic spinal cord (Figure 2). Pan computed tomography (CT) scan and bronchoscopy were also performed and were unremarkable for malignancies.

On the second day of admission, the patient received 1 g of intravenous methylprednisolone with a presumptive diagnosis of MS relapse. Unexpectedly, the patient deteriorated 5 h after receiving the dose. He initially complained of nausea, which was followed by choking on liquids, worsening numbness on the left side, and followed by inability to swallow his saliva. The pupils were 7 mm and non-reactive bilaterally, and there was decreased facial sensation on the left side associated with a head drop. The power was normal in all limbs except for the left upper limb, which was 3 of 5. Reflexes were absent, and the plantar response was equivocal. Then, the patient was intubated and transferred to the intensive care unit (ICU) on the third day of admission. In the ICU, a central line was placed in the internal jugular vein, and plasma exchange was started. Replacement fluid consisted of fresh frozen plasma and normal saline in equal volumes. Plasma exchange sessions were done on a daily basis; after 8 days, improvement in the pupil size and eye movement has been noticed, and we have decided to add two more sessions. During the treatment period, the patient did not experience any complications related to the replacement fluid or vascular access, and the total volume of plasma exchanged was 3,600 cc.

Following the deterioration of the patient, MRI with contrast of the brain and whole spine was performed and showed no new changes compared with the MRI findings performed on admission. A follow-up MRI was done 10 days on the 13th day of admission and revealed no changes compared with the previous one.

On the third day, visual evoked potential was performed and revealed a left P100 wave latency prolongation: left, 170 ms; right, 191 ms. Nerve conduction study (NCS) was performed and showed normal findings. However, the NCS's interpretation was limited by ICU artifacts. Additionally, lumbar puncture was repeated on the fourth day of admission and showed 0 white blood cells, and the protein level of 71 mg/dL, which showed typical albuminocytologic dissociation.

Further diagnostic tests were ordered and showed the following: Testing for anti-ganglioside antibodies revealed positivity for anti-GQ1b with titers of 324. Anti-NMO antibodies, anti-MOG antibodies, anti-VGKC, GlyR, anti-neurofacin antibodies, botulism toxin, and paraneoplastic panel were all negative. Antibodies against *Campylobacter jejuni* were negative.

The patient was in the ICU for 18 days; during which, he developed ventilator-associated pneumonia and was managed with antibiotics. Tracheostomy was performed 10 days after ICU admission due to failure of weaning off intubation.

Electromyography and NCS were repeated on the 25th day of admission. The NCS of the left and right median, ulnar, peroneal, and tibial nerves revealed normal distal latencies, compound muscle action potentials (CMAPs), and conduction velocities. Electromyography detected changes in enervation in the form of fibrillation and positive sharp waves in the right frontalis and orbicularis oris muscles with rapidly firing motor unit action potentials (MUAPs), which showed reduced recruitment.

The tracheostomy weaning-off protocol was started soon after transfer to the ward. The nasogastric tube was removed with closure of the tracheostomy, and the patient tolerated a regular diet. On the 40th day, the patient was cleared for discharge as he was able to feed himself independently and ambulate by using a walker.

On the 66th day, the patient visited the clinic, was able to walk without any assistance, and has reported significant improvement in ataxia and diplopia. On examination, the right pupil was 4 mm reactive, and the left pupil was 5 mm with a sluggish reaction to light. Additionally, there was a mild limitation in the vertical gaze and adduction movement of both eyes. Muscle tone was normal, and power was 5/5 in the right upper and lower limbs, and 4/5 in the left upper limb. Deep tendon reflexes were +1 in the right and left upper limbs and absent in the lower limbs. The neck flexors and extensors had a power of 5/5. There was a noticeable improvement in the finger-to-nose test; however, dysmetria was noted more on the left side. The patient can walk unassisted with mild ataxia and can perform tandem gait with moderate difficulty. After almost 7 months from discharge, the brain MRI with contrast was repeated and showed a stable demyelinating process with no new T2 hyperintense lesions or enhancement.

## Discussion and Conclusions

Our patient fulfills the MRI McDonald criteria for dissemination in space along with the presence of OCBs; however, the diagnosis of MS could not be made as the patient did not exhibit a clear clinical demyelinating attack as the numbness was intermittent, lasting only for a few seconds. Additionally, the numbness could be explained as a part of Miller Fisher's initial symptoms. Furthermore, the INO is most likely a pseudo-INO rather than a true one, as the site and the size of the lesion seen in the pons do not explain the occurrence of a true INO.

During the course of his hospital stay, the patient exhibited the typical triad of MFS, consisting of ataxia, areflexia, and ophthalmoplegia. Another finding supporting the diagnosis of MFS was the presence of albuminocytologic dissociation in the second CSF sample of the patient. GQ1b antiganglioside antibody, which is associated with MFS, was also positive, with a titer of 324 (4).

Our differentials steered away from MS relapse and toward other PNS disorders, following rapid deterioration after methylprednisolone administration. Guillain-Barre syndrome was considered as one of the differential diagnoses; however, the clinical picture of the patient in the form of ophthalmoplegia and ataxia did not support the diagnosis. Furthermore, the absence of deep tendon reflexes and the lack of alteration in the mental status of the patient during the course of the disease argued against Bickerstaff encephalitis. Another differential was botulism; however, it was relatively unlikely because of its rarity and unremarkable history of recent ingestion, which was objectively ruled out by the negative result of the botulism toxin test.

Currently, there is no established diagnostic criteria for combined central and peripheral demyelination (CCPD) (5). The patient was labeled as a case of CCPD due to the involvement of both CNS and PNS. A study performed by Pender et al. in 2003 showed that patients with primary progressive MS have a significant increase in peripheral T-cell reactivity to GM3 and GQ1b gangliosides compared with healthy subjects and patients with other CNS diseases, which might explain the co-occurrence of MFS and the characteristic MS lesions in our patient (6). The absence of enhancing lesions and the possibility of having transient OCBs in the CSF makes it unlikely that the patient is having the two conditions simultaneously. For that, further follow-up is needed for possible future relapses and recurrences to confirm the diagnosis of MS. Managing this patient was challenging since there was no previous treatment guide for a similar course of illness to predict the outcomes.

We searched PubMed and other databases with the following search words: “miller fisher,” “central nervous system,” and “magnetic resonance imaging.” Multiple studies describing patients diagnosed with MFS with a concurrent CNS lesion have been found (7–10); however, the clinical course, CSF results, and MRI features in these patients were not typical for MS (Table 1). In a case reported by Xu and Liu a 37-year-old man presented with diplopia, dizziness, ptosis, and bilateral upper limb numbness (7). Through investigation of the patient, MRI revealed multiple lesions at the juxtacortex, subcortex, and deep white matter in the left frontal and occipital lobe, the signals were hypointense in T1-weighted images and isointense in diffusion-weighted images, and there was no enhancement after contrast administration. CSF analysis was significant for pleocytosis alone. The NCS was normal, apart from the absent tibial H reflexes. During the stay of the patient, no antibodies were detected. The patient was managed with IVIG (0.4 g/kg per day for 5 days) and recovered fully after 60 days. The mentioned case had an almost identical initial clinical presentation to our patient, in addition to being male in the same age group. However, there was no diagnosis of CNS lesions, and findings of our patient did not return to the baseline after 66 days and 10 sessions of plasma exchange.

**Table 1:** Reported cases of Miller-Fisher syndrome with concurrent central nervous system lesions.

**References**	**Present case**	**Xu and Liu (7)**	**Tezer et al. (8)**	**Echaniz-Laguna et al. (9)**	**Urushitani et al. (10)**
Age	31	37	54	42	50
Sex	M	M	M	F	M
Initial symptoms	Diplopia, dysarthria, left sided numbness, unsteadiness, and constipation	Diplopia, unsteady gait dizziness, left eyelid ptosis and distal numbness on both upper limbs	Vertigo, diplopia, difficulty in swallowing, and unsteadiness	Diplopia, unsteadiness, and lower limb weakness	Diplopia, bilateral eyelid ptosis, ataxic gait, nausea, and vomiting
Albuminocytological dissociation	Yes	No	No	Yes	Yes
CSF OCB	Detected	Not reported	Negative	Negative	Not reported
NCS	Abnormal	Abnormal	Abnormal	Abnormal	Normal
Antibodies profile	GQ1b	Negative	Not tested	Negative	Not tested
MRI findings	Brain: multiple lesions in cortical, juxtacortical, subcortical, periventricular, and pons Spine: multiple cervical and thoracic intramedullary lesions	Brain: multiple lesions in the juxtacortex, subcortex and deep white matter	Lesions in the pons, medulla oblongata and cerebellar peduncle	Lesions in cerebral white matter, brainstem, and cerebellum	Enhancing lesions in the spinocerebellar tracts at the level of the lower medulla
Treatment	Plasma exchange (10 sessions)	IVIG (1 course)	IVIG (1 course) Acyclovir	IV methylprednisolone	IV methylprednisolone plasma exchange
Outcome	Partial recovery after 66 days	Complete recovery after 60 days	Complete recovery after 6 months	Complete recovery after 40 days	Complete recovery after 3 months

*CSF, cerebrospinal fluid; OCB, oligoclonal bands; NCS, nerve conduction study; IVIG, IV immunoglobulin*.

In a case report published in 1995, a 50-year-old man presented with diplopia, bilateral ptosis, and ataxic gait (10). MRI revealed bilaterally enhancing lesions in the lateral lower medulla, consistent with the anterior and posterior spinocerebellar tracts. The patient was diagnosed with MFS, managed with methylprednisolone and plasma exchange, and had full recovery within 3 months. As shown in Table 1, all cases reported to have MFS with CNS lesions achieved full recovery; moreover, their recovery period was variable and ranged from 40 days to 6 months.

Compared with studies reporting on CCPD cases, a case reported by Katchanov et al. where they describe a patient diagnosed with acute disseminated encephalomyelitis and acute inflammatory demyelinating polyradiculoneuropathy, the laboratory workup was significant for a positive GQ1b and GM1; he was managed with methylprednisolone, and the condition worsened; following which, he received one course of IVIG, followed by six sessions of plasma exchange and had partial recovery within 1 year (11). Another patient diagnosed with clinically isolated syndrome and chronic inflammatory demyelinating polyradiculoneuropathy (CIDP) simultaneously was managed with prednisone 100 mg one time daily and azathioprine 100 mg two times daily and achieved partial recovery within 6 months (12). Moreover, a case reported in 2019 with an unspecified disease of the CNS accompanied with CIDP had a positive anti-neurofacin, which is associated with CCPD, and it has been recently found that patients with positive anti-neurofacin respond well to IVIG or plasma exchange (5, 13). Although our patient had a negative anti-neurofacin during the investigation, he responded well to plasma exchange, which may indicate that plasma can be still beneficial in cases with CCPD and negative anti-neurofacin. As for the case mentioned earlier, he was managed with IVIG and motor rehabilitation, and there was a good response to treatment for 3 months; after which, the condition of the patient worsened; after which, he received methylprednisolone. Then, he had relapsed and was prescribed azathioprine and steroids, and partial recovery was achieved after 3 years. In a case reported by Nouha et al., the patient was diagnosed with MS and chronic inflammatory demyelinating polyradiculoneuropathy (CIDP) (14). They managed him with methylprednisolone and interferon β-1a; however, the treatment resulted in only mild initial improvement and was ineffective. Regarding the management of CCPD cases, plasma exchange was found to be effective in 87.5% of the cases, and it was, indeed, effective in this case (15). Although recovery periods varied between patients diagnosed with CCPD, none of the reported cases we accessed gained full recovery.

This case sheds light on a different combination of CNS and PNS involvement consisting of RIS and MFS, which warrants extensive investigation in CCPD cases, which is now generally viewed as a combination of MS and CIDP alone. Further studies and analysis of available data are needed with respect to this topic since the resources and cases shared are insufficient to have a clear guideline for their management or follow-up to determine the possible prognosis.

## Data Availability Statement

The original contributions presented in the study are included in the article/Supplementary Material, further inquiries can be directed to the corresponding author/s.

## Ethics Statement

Written informed consent was obtained from the individual(s) for the publication of any potentially identifiable images or data included in this article.

## Author Contributions

All authors listed have made a substantial, direct and intellectual contribution to the work, and approved it for publication.

## Conflict of Interest

The authors declare that the research was conducted in the absence of any commercial or financial relationships that could be construed as a potential conflict of interest.

## Publisher's Note

All claims expressed in this article are solely those of the authors and do not necessarily represent those of their affiliated organizations, or those of the publisher, the editors and the reviewers. Any product that may be evaluated in this article, or claim that may be made by its manufacturer, is not guaranteed or endorsed by the publisher.

## Abbreviations

CCPD: Combined central and peripheral demyelination
RIS: radiologically isolated syndrome
CNS: central nervous system
MFS: miller-fisher syndrome
PNS: peripheral nervous system
MS: multiple sclerosis
CSF: cerebrospinal fluid
OCB: oligoclonal bands
MRI: magnetic resonance imaging
CT: computed tomography
ICU: intensive care unit
NCS: nerve conduction study
CMAPs: compound muscle action potential
MUAPs: motor unit action potentials
CIDP: chronic inflammatory demyelinating polyradiculoneuropathy.
