# Supplementary material for: A New Report of Combined Central and Peripheral Demyelination: A Case Report
Source: Front Neurol. 2021 Nov 19;12:730129. doi: 10.3389/fneur.2021.730129 (PMC8639527; doi:10.3389/fneur.2021.730129)
Supplement: Supplementary file 1 [file Data_Sheet_1.PDF]

CARE case report guidelines

CARE Checklist of information to include when writing a case report

(cc) BY-NC-ND

Topic	Item	Checklist item description	Reported on Line
**Title**	1	The diagnosis or intervention of primary focus followed by the words "case report"	Title
**Key Words**	2	2 to 5 key words that identify diagnoses or interventions in this case report, including "case report"	28
**Abstract (no references)**	3a	Introduction: What is unique about this case and what does it add to the scientific literature?	23
	3b	Main symptoms and/or important clinical findings	21
	3c	The main diagnoses, therapeutic interventions, and outcomes	24
	3d	Conclusion—What is the main "take-away" lesson(s) from this case?	24
**Introduction**	4	One or two paragraphs summarizing why this case is unique (**may include references**)	30
**Patient Information**	5a	De-identified patient specific information.	43
	5b	Primary concerns and symptoms of the patient.	44
	5c	Medical, family, and psycho-social history including relevant genetic information	43
	5d	Relevant past interventions with outcomes	47
**Clinical Findings**	6	Describe significant physical examination (PE) and important clinical findings.	52
**Timeline**	7	Historical and current information from this episode of care organized as a timeline	77
**Diagnostic Assessment**	8a	Diagnostic testing (such as PE, laboratory testing, imaging, surveys).	61
	8b	Diagnostic challenges (such as access to testing, financial, or cultural)	N/A
	8c	Diagnosis (including other diagnoses considered)	125
	8d	Prognosis (such as staging in oncology) where applicable	N/A
**Therapeutic Intervention**	9a	Types of therapeutic intervention (such as pharmacologic, surgical, preventive, self-care)	N/A
	9b	Administration of therapeutic intervention (such as dosage, strength, duration)	76
	9c	Changes in therapeutic intervention (with rationale)	83,125
**Follow-up and Outcomes**	10a	Clinician and patient-assessed outcomes (if available)	109
	10b	Important follow-up diagnostic and other test results	86
	10c	Intervention adherence and tolerability (How was this assessed?)	N/A
	10d	Adverse and unanticipated events	77
**Discussion**	11a	A scientific discussion of the strengths AND limitations associated with this case report	136
	11b	Discussion of the relevant medical literature **with references**.	135
	11c	The scientific rationale for any conclusions (including assessment of possible causes)	132
	11d	The primary "take-away" lessons of this case report (without references) in a one paragraph conclusion	180
**Patient Perspective**	12	The patient should share their perspective in one to two paragraphs on the treatment(s) they received	N/A
**Informed Consent**	13	Did the patient give informed consent? Please provide if requested	Yes ☑ No ☐
